# Fabrication of Ni–Cu–W Graded Coatings by Plasma Spray Deposition and Laser Remelting

**DOI:** 10.3390/ma15082911

**Published:** 2022-04-15

**Authors:** Jie Liao, Liangbo Zhang, Cong Peng, Yandong Jia, Gang Wang, Hui Wang, Xuguang An

**Affiliations:** 1 Institute of Materials, Shanghai University, Shanghai 200444, China; lj1992972265@163.com (J.L.); liangbo0703@shu.edu.cn (L.Z.); pengcong@shu.edu.cn (C.P.); g.wang@shu.edu.cn (G.W.); 2 Zhejiang Institute of Advanced Materials, Shanghai University, Jiaxing 314113, China; 3 Interdisciplinary Materials Research Center, Institute for Advanced Study, Chengdu University, Chengdu 610106, China; qinghe5525@163.com (H.W.); anxuguang@cdu.edu.cn (X.A.)

**Keywords:** graded coating, atmospheric plasma spraying, laser remelting, corrosion resistance

## Abstract

In this study, Ni–Cu–W graded coatings are produced by atmospheric plasma spraying and subsequently remelted by laser. The surface morphology, hardness, compositional fluctuations and corrosion resistance of the Ni–Cu–W coating are investigated. The coatings after laser remelting are densified and become more homogenous with an excellent corrosion resistance and high hardness, which can be used to explore the new materials.

## 1. Introduction

In order to achieve the goal of halving the research and development time and cost of advanced materials, it is imperative to develop high-throughput preparation technology for the preparation and screening of new materials. Exploring and developing a combination material chip, which is suitable for the rapid and efficient preparation of multiple composition gradients, has become a steadily growing method in the current research and development process of advanced materials [1,2]. Ni–Cu alloys are widely used due to their excellent properties, such as marvelous corrosion resistance, high oxidation resistance, and magnetic properties, etc. Traditionally, it is considered that Ni contributes to good ductility, corrosion resistance, and ferro-magnetism, meanwhile, Cu improves fantabulous conductivity [3]. As a typical refractory metallic element with a high melting temperature (3407 °C), W exhibits high hardness. Hence, some binder elements with low melting temperatures, such as Ni, Cu, and Fe [4,5,6], are normally added into W to form dense structures. W–Ni alloys are considered as a potential environment-friendly material for the replacement of hard Cr alloys due to their excellent hardness, wear resistance, desirable anticorrosion properties, electro-catalytic features, etc. Zhang et al. [7] investigated the effects of Ni content on the microhardness and microstructure evolution of a W–Ni alloy synthesized by selective laser melting (SLM). It was found that a combination of liquid-phase sintering (LPS) and partially melting of W particles could produce a W–Ni alloy. Wang et al. [8] studied the influence of SLM parameters on the microstructures and the metallurgical mechanisms of W-10Ni-10Cu (at.%) fabricated by SLM equipped with a 500 W fiber laser. In order to further widen the application of Ni–Cu–W alloys, it is imperative to explore alloys with new compositions. 

The graded coatings, fabricated by plasma spraying that is often used to deposit relatively thick coatings [9,10], have been widely used in many fields to prevent metallic materials from wearing [11], corrosion [12] and oxidizing [13]. Nevertheless, the plasma spraying process usually brings out disadvantages, such as insufficient bonding strength between the coating and the substrate, which would result in peeling off of the coatings due to a high residual stress [14]. Furthermore, pores, cracks, and semi-melted particles embedding in the coatings would lead to low hardness, poor wear, and anti-corrosion performances [15]. Thus, post-treatments, such as laser remelting, may be carried out to optimize the microstructure and properties of coatings fabricated by plasma spraying. During the laser remelting process, the laser beam can melt the surface without adding any metal elements, which can significantly eliminate the pores and cracks in the coatings [16,17,18,19,20]. Wang et al. [21] studied the effects of laser remelting on cobalt-based and nickel-based alloy plasma sprayed coatings. The results showed that laser remelting can significantly improve the density and distribution of the microstructure of the plasma sprayed coating. Moreover, the defects were reduced. Both abrasion resistance and corrosion resistance were significantly improved after laser remelting. 

Our previous studies [22] reported the successful fabrication of Ni-Al graded coating with excellent performances by using plasma spraying and laser remelting. Based on this, in the present study, a combinatorial technology based on plasma spray deposition and laser remelting was used to fabricate the Ni–Cu–W graded coatings. The components of Ni–Cu–W coatings were optimized by a high-throughput fabrication technique based on plasma spraying. Then, the relationships among microstructures, hardness and electrical conductivity were investigated. 

## 2. Experimental Section

**Figure 1:**
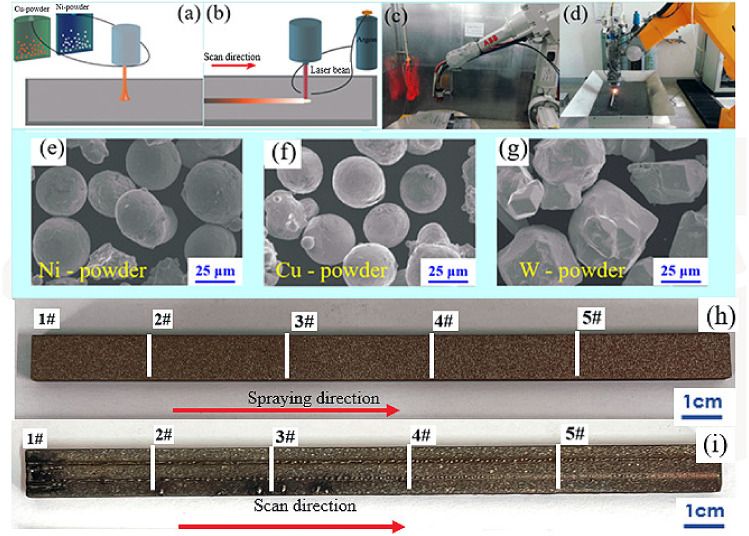
The schematic diagram illustrating the: (**a**) plasma spray deposition system, and (**b**) the laser remelting equipment, (**c**) Plasma spraying experimental equipment, and (**d**) Laser remelting experimental equipment, (**e**–**g**) Scanning electron microscopy images (SEM) showing the morphology of the powders. (**h**) Camera images of the entire coated surface and (**i**) remelted surface.

In the current study, a Ni-plate was used as a substrate for the plasma spraying. The size of the Ni-plate was 150 mm × 10 mm × 5 mm. Cu, Ni and W powders (≥99.9% purity) with particle sizes varied from 35 to 80 μm were used as feedstocks. The Ni–Cu–W graded coatings were deposited in an XM-100 spraying gun by an atmospheric plasma spraying (APS) system (XM-80SK, SHXM-PT, Shanghai, China). The APS schematic diagram and equipment diagram were shown in Figure 1a,c. The plasma spraying parameters for deposition were as follows: (a) current was 450 A, (b) voltage was 56.2 V, (c) primary Ar gas flow was about 45 L/min, flow rate of auxiliary gas H_2_ was 3 L/min and (d) spraying distance was about 110 mm. Ni–W mixed powders and Cu powders were stored in two different powder jets of the multi-station intelligent powder delivery system, respectively. The powder feed rates of both jets were controlled by the variation of the rotational speed, and finally realized the preparation of gradient coating. Figure 1h shows the images of the coated surface. After obtaining the depositions, the surface of as-sprayed coatings was remelted utilizing the Ytterbium Laser System with a wavelength of λ = 1.06 µm. The laser remelting schematic diagram and equipment diagram were shown in Figure 1b,d. The laser output power was 450 W, and the scanning velocity was 4 mm/s. Meanwhile, Ar was used as the protective gas argon to prevent the surface of the coating from being oxidized. Figure 1g demonstrates the macro morphology of the entire remelted surface. Figure 1e–g shows the morphologies of the three-feedstock powders, which were observed in scanning electron microscope (SEM) (SU-1510, Hitachi, Tokyo, Japan) operated at 15 kV. The powders were heated in the oven at 150 °C for 1.5 h before spraying. Prior to deposition, the substrates were blasted by grit in order to reinforce the adherence of coating surface and were degreased by ultrasonic cleaning in acetone and dried in a drying oven at 150 °C for 1.5 h. 

The microstructures of the coating before and after laser remelting were observed by SEM equipped with an X-ray energy dispersive spectroscopy (EDS). The phases of the coating before and after laser remelting were analyzed by an X-ray diffraction micrometer (XRD, D/MAX-3BX, Rigaku, Tokyo, Japan) with a Cu-Kα radiation operated at 40 kV from 20° to 100°, and a scanning speed of 4°/min. The hardness was measured using Vickers Indenter applying a continuous load of 2 N (Duramin-40, Struers, Copenhagen, Denmark). The penetration depth of the indentation was limited to be less than 10% of the film thickness to avoid the substrate effect. The corrosion behavior was investigated by electrochemical workstation (CHI660C, Shanghai, China) in 3.5 wt.% NaCl solution at room temperature. A steady open-circuit potential was obtained after the specimens were immersed in NaCl for 1 h. The potentiodynamic polarization tests were carried out at a scanning rate of 1 mV/s, and each specimen was tested at least three times to secure reproducibility.

## 3. Results and Discussion

**Figure 2:**
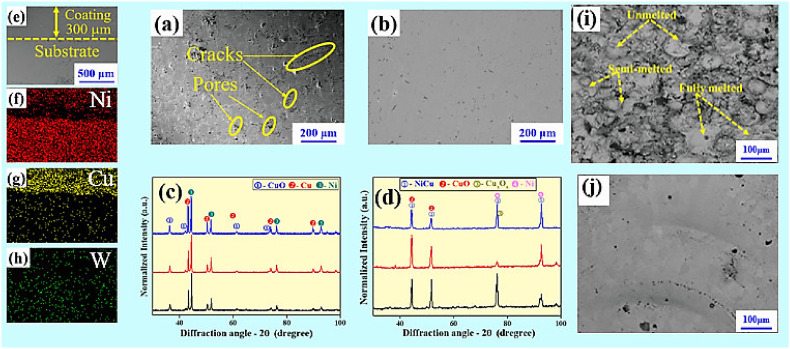
(**a**) The morphologies of coatings before and (**b**) after remelting, (**c**) X-ray diffraction spectrum of the coatings before and (**d**) after remelting, (**e**) The cross-sectional morphology of the coating; (**f**–**h**) the elemental distributions map at the junction of the coating and substrate, surface microstructure of the coating after plasma spraying (**i**) and laser remelting (**j**).

Figure 2a,b depict the morphologies of coatings before and after remelting. It was found that the surface of coating prepared by plasma spraying exhibits many pores, cracks and semi-melted particles. However, the defects on the surface after laser remelting decrease obviously, and the surface demonstrates smooth and homogeneous characteristics. This can be ascribed to the high-energy laser beam and the formation of a molten pool. Figure 2i,j show the surface microstructure of coatings before and after laser remelting. It was found that the surface of the sprayed coating presented some fully-molten, semi-molten, and unmelted particles [23]. The surface roughness of the coated samples after laser remelting treatment was reduced, and there were no obvious unmelted or semi-melted particles. The XRD patterns for the same region on the as-sprayed and remelted specimen are shown in Figure 2c,d. The region characterized by XRD was deposited by a composition containing a Ni:Cu ratio of 2:1 in weight, and the content of W being 1 wt.%. For the as-spraying coating, the XRD pattern mainly shows the metallic Ni, Cu and CuO phases. After remelting, the diffraction peaks corresponding to Cu disappear, and a NiCu and a CuO phases are formed, which indicates that alloying between Ni and Cu occurs. Figure 2e presents the cross-sectional morphology of the coating with a thickness of approximately 300 µm. Figure 2f–h show the elemental distribution maps for the junction area between the coating and substrate, which prove that the three elements are evenly distributed. 

**Figure 3:**
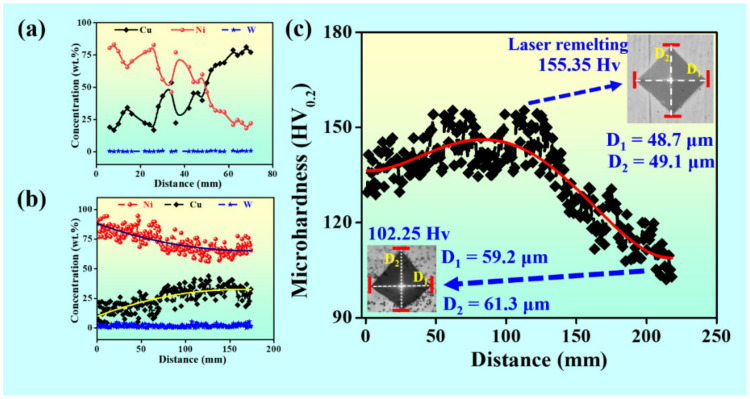
(**a**) The compositional variation of the coating before and (**b**) after laser remelting, and (**c**) the hardness of the coating after laser remelting.

Figure 3a shows the elemental fluctuations along the gradient direction. With W percentage being constant, it has been found that Ni content increases with the decrease in Cu content. Before laser remelting, the Ni content varies from 80% to 20%, and the Cu content transforms from 20% to 80%. The coating was divided into five sections along the gradient direction of decreasing Ni content, which are numbered as Sample 1# (Ni: 65–77 wt.%, Cu: 22–34 wt.%, W: 0.1–1 wt.%), 2# (Ni: 55–65 wt.%, Cu: 34–44 wt.%, W: 0.1–1 wt.%), 3# (Ni: 44–55 wt.%, Cu: 44–55 wt.%, W: 0.1–1 wt.%), 4# (Ni: 34–44 wt.%, Cu: 55–67 wt.%, W: 0.1–1 wt.%), 5# (Ni: 20–34 wt.%, Cu: 67 wt.% 80 wt.%, W: 0.1–1 wt.%). Due to the reduction in defects and the subsequent homogenization after laser remelting, as shown in Figure 3b, the Ni content and the Cu content changes from 80% to 60%, and from 20% to 36%, respectively. Figure 3c shows the hardness distribution of the coating after laser remelting. It can be seen that the hardness varies from 102 to 155 HV0.2. With the compositional fluctuations, the hardness of the graded coating also varies directionally. The tendency of the hardness value initially increases and subsequently decreases. The peak hardness is observed at the sample 2#, which may be due to the appearance of the Ni–W phase [24]. With enriching the Cu element and depleting the Ni content, the hardness shows a decreasing trend.

**Figure 4:**
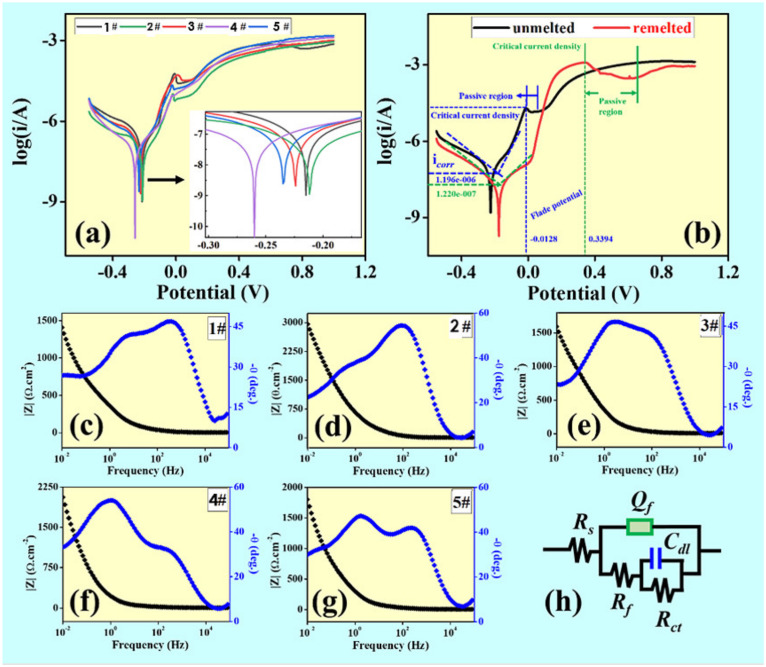
(**a**) potentiodynamic polarization curves of 1#, 2#, 3#, 4# and 5# as-sprayed coatings in 3.5% NaCl solution, (**b**) potentiodynamic polarization curves of coatings before and after remelting, (**c**–**g**) the bode plots of 1#, 2#, 3#, 4# and 5# coatings in the 3.5 wt.% NaCl solution at 25 °C after 1 h of immersion, (**h**) the equivalent circuits of EIS for coatings in the 3.5 wt.% NaCl solution.

It is observed that the coatings (samples 1#, 2#, 3#, 4# and 5#) exhibit different corrosion resistance due to the different contents of Ni and Cu. The potentiodynamic polarization curves of five specimens in the NaCl solution are shown in Figure 4a. We can see that the samples 1# and 2# have higher corrosion potential than other samples. The electrochemical impedance spectroscopy (EIS) plots of the passive film formed in the NaCl solution at 25 °C after being immersed for 1 h are exhibited in Figure 4c–g. In the Bode plots, the value of |Z| of the 2# coating is ~3000 Ω·cm^2^, which is higher than those of the 1# (~1408 Ω·cm^2^), 3# (~1595 Ω·cm^2^), 4# (~2601 Ω·cm^2^) and 5# (~1805 Ω·cm^2^), indicating that the corrosion resistance of the 2# coating is superior to the other four coatings in 3.5 wt.% NaCl solution. Furthermore, the maximum phase angle (~54.4) of the 2# coating was higher than those of the 1# (~46.9), 3# (~46.6), 4# (~54.1) and 5# (~47.2) coatings, suggesting that there is a great improvement in corrosion property. The equivalent circuit of the coatings used for fitting the EIS datum of the 1#, 2#, 3#, 4# and 5# coatings in the 3.5 wt.% NaCl solution is presented in Figure 4h. In the equivalent circuit, Rs is the resistance of solution, Qf is the capacitances of film, Rf is the resistance of the film, Rct is the resistance of the passive film, and Cdl is a double-charge layer. The parameter of Cdl represents the surface heterogeneity, reflecting the compactness of passive film.

Figure 4b shows the polarization curves of the unmelted area and the remelted area about 10 mm in length on the same coating. Compared with the spraying coating, the corrosion potential of the laser remelting coating increases, with decreasing corrosion current density. This indicates that the corrosion tendency of the laser remelted coating is weakened, as the corrosion rate is reduced (the corrosion resistance improved). In addition, compared with spraying coating, the polarization curve after laser remelting displays an apparent passivation area. The protective film formed by passivation can effectively prevent the corrosive medium from penetrating up to the substrate, which is beneficial to improve the corrosion resistance of the coating. The corrosion products are observed on the surface of the coating, thereby isolating the coating from further contact with the corrosive medium to a certain extent, thereby slowing down the corrosion rate. Laser remelting can promote the homogenization of the coating composition, and significantly eliminate the layered structure and pores of the spraying coating in particular, thereby reducing the rate of non-uniform corrosion.

**Figure 5:**
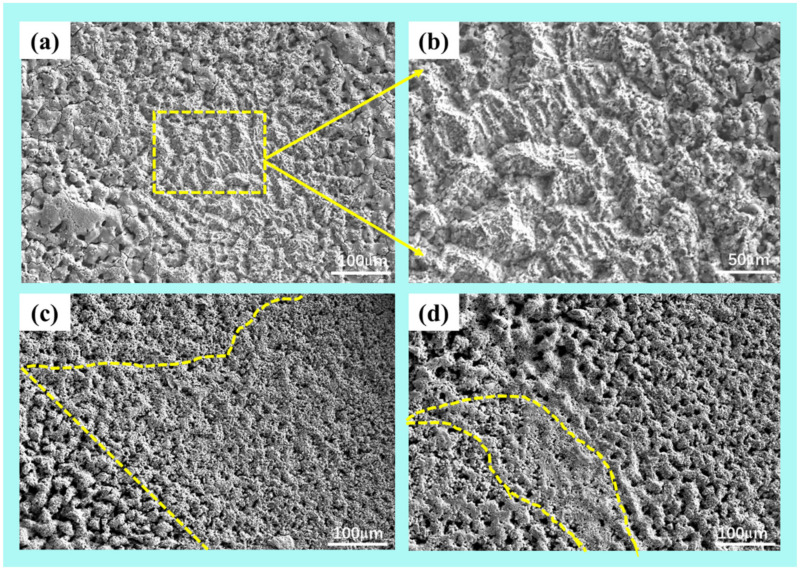
Surface microstructures of the coatings after plasma spraying tested in 3.5 wt.% NaCl solution after 1 h of immersion: (**a**) 2#; (**b**) higher magnification image, (**c**) 3#, (**d**) 4#.

Figure 5 shows the surface microstructures of the coatings of plasma spraying tested in 3.5 wt.% NaCl solution after 1 h of immersion. According to the measured electrochemical polarization curves of five samples, the coatings of 2#, 3#, and 4# samples were selected and the surface morphology was characterized. Usually Cl- in NaCl solution is the main cause of corrosion pits. From Figure 5a, it can be found that there are few pitting pits on the coating surface of the 2# (Ni: 55–65 wt.%, Cu: 34–44 wt.%, W: 0.1–1 wt.%) sample, and the microstructure of the passivation film is shown in Figure 5b. There are no large pitting pits, but the surface is undulating and uneven. For the 3# sample, as the immersion time increases, pitting corrosion occurs first, and then the passive film formed will gradually be eroded. The corrosion pits on the coating surface of the 4# sample are obvious, the corrosion rate is the highest, and the corrosion resistance is the worst.

**Figure 6:**
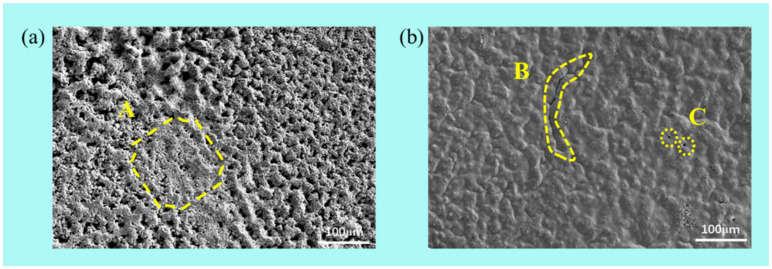
Surface microstructures of the coating after plasma spraying (**a**) and laser remelting (**b**) tested in 3.5 wt.% NaCl solution after 1 h of immersion.

Figure 6 shows the surface microstructure of the coating after plasma spraying and laser remelting after immersing in 3.5% NaCl solution for 1 h. It can be seen from Figure 6a that a passivation film is formed on the surface of the sample coating after corrosion, but there are many pitting pits of different sizes on the surface of the passivation film. The passivation film formed in area A is dense. Whereas, due to the existence of defects, a passivation film will be formed on the surface of the coating to reduce the corrosion rate. Meanwhile, because of the existence of corrosion pits on the surface, as the corrosion pits gradually become larger, the passivation film is quickly broken down. Figure 6b shows the surface of the coating corroded by the laser remelting process. It can be found that there are fewer pitting pits on the surface of the coating sample, which makes the coating passivation time longer and reduces the corrosion rate of the coating. Defects such as microcracks and pores still presented on the coating surface, as shown in areas B and C in Figure 6b, making the NaCl solution gradually corroded from the defects.

## 4. Conclusions

The Ni–Cu–W graded coatings were successfully fabricated by a combinatorial technology based on plasma spray deposition and laser remelting. In addition, the pure metal phases (Ni and Cu phases) in the plasma spraying coatings transform to the Ni–Cu phase. A relatively superior composition (Ni: 55–65 wt.%, Cu: 34–44 wt.%, W: 0.1–1 wt.%) is screened according to the hardness measurement, and the result of corrosion resistance. Therefore, it is possible to promptly and effectively explore the new compositions, structures, and mechanical properties of materials by plasma spraying accompanied with laser remelting, which can accelerate the process of screening new materials in the future.

## Data Availability

Data are contained within the article.
